# Thalamocortical Connectivity and Microstructural Changes in Congenital and Late Blindness

**DOI:** 10.1155/2017/9807512

**Published:** 2017-03-13

**Authors:** N. H. Reislev, T. B. Dyrby, H. R. Siebner, H. Lundell, M. Ptito, R. Kupers

**Affiliations:** ^1^Danish Research Centre for Magnetic Resonance, Centre for Functional and Diagnostic Imaging and Research, Copenhagen University Hospital Hvidovre, Hvidovre, Denmark; ^2^Department of Applied Mathematics and Computer Science, Technical University of Denmark, Kongens Lyngby, Denmark; ^3^Department of Neurology, Copenhagen University Hospital Bispebjerg, Copenhagen, Denmark; ^4^Laboratory of Neuropsychiatry, Psychiatric Centre Copenhagen, Copenhagen, Denmark; ^5^School of Optometry, Université de Montréal, Montréal, QC, Canada; ^6^BRAINlab, Danish Centre for Sleep Medicine, Rigshospitalet, Department of Clinical Neurophysiology, University of Copenhagen, Copenhagen, Denmark; ^7^Department of Radiology & Biomedical Imaging, Yale University, 300 Cedar Street, New Haven, CT 06520, USA

## Abstract

There is ample evidence that the occipital cortex of congenitally blind individuals processes nonvisual information. It remains a debate whether the cross-modal activation of the occipital cortex is mediated through the modulation of preexisting corticocortical projections or the reorganisation of thalamocortical connectivity. Current knowledge on this topic largely stems from anatomical studies in animal models. The aim of this study was to test whether purported changes in thalamocortical connectivity in blindness can be revealed by tractography based on diffusion-weighted magnetic resonance imaging. To assess the thalamocortical network, we used a clustering method based on the thalamic white matter projections towards predefined cortical regions. Five thalamic clusters were obtained in each group representing their cortical projections. Although we did not find differences in the thalamocortical network between congenitally blind individuals, late blind individuals, and normal sighted controls, diffusion tensor imaging (DTI) indices revealed significant microstructural changes within thalamic clusters of both blind groups. Furthermore, we find a significant decrease in fractional anisotropy (FA) in occipital and temporal thalamocortical projections in both blind groups that were not captured at the network level. This suggests that plastic microstructural changes have taken place, but not in a degree to be reflected in the tractography-based thalamocortical network.

## 1. Introduction

There is strong evidence that the occipital cortex in visually deprived humans undergoes massive cross-modal reorganisation and processes sensory information from other modalities than vision (for review, see [1]). However, there is no consensus as to whether these cross-modal responses are mediated by structural alterations in corticocortical or thalamocortical connectivity [2, 3], or a combination of both [4].

The thalamus forms an important relay between sensory input and the cerebral cortex for all sensory modalities, with the exception of olfaction. Specialised nuclei within the thalamus receive and process specific sensory input which is further sent to specialised cortical areas via thalamocortical projections [5, 6]. In the normal healthy human brain, visual information is relayed from the lateral geniculate nucleus (LGN) to the primary visual cortex via the optic radiations (for review, see Metzger et al. [7]).

In blind individuals, cross-modal responses in the occipital cortex could potentially be mediated through rerouting of information from auditory and somatosensory thalamic nuclei to the occipital cortex via the LGN. Although both the LGN and the optic radiations are reduced in size in congenital blindness [8], nonvisual information might be relayed to the visual cortex through the optic radiations [9–12]. This would imply the formation of novel ectopic connections between the thalamic somatosensory nucleus and the LGN, as has been shown in animal models of blindness [13, 14]. Alternatively, nonvisual occipital activation could also be mediated by strengthening of existing corticocortical connections [15]. To the best of our knowledge, no studies have reported on the contribution of the thalamus to the transfer of nonvisual information to the visual cortex in human blind subjects. Therefore, purported alterations in thalamocortical connectivity in the human blind brain remain to be proven.

The aim of this study is therefore to map thalamocortical structural connectivity in vivo using tractography based on diffusion-weighted imaging (DWI). A second aim was to test whether changes in the intrinsic regional microstructure of the thalamic nuclei can be demonstrated by diffusion tensor imaging (DTI). Adopting the method of Behrens et al. [16], we segmented the thalamus into clusters based on their thalamocortical connectivity as revealed by DWI-based tractography and compared the structural thalamocortical connectivity patterns among age-matched groups of congenitally blind (CB), late blind (LB), and normal sighted (NS) control subjects. We reasoned that between-group changes in thalamocortical white matter projections would be reflected by altered thalamic segmentation patterns and that these alterations would depend on the onset of blindness.

## 2. Materials and Methods

### 2.1. Subjects and Data Acquisition

A total of 12 CB (mean age 42 ± 13 years), 15 LB (mean age 52 ± 15 years, mean onset of blindness 16.6 ± 8.9 years), and 15 NS (mean age 46 ± 13 years) individuals were included in the study. The cause of blindness was restricted to be of peripheral origin and subjects with residual vision were excluded. The Danish ethics committee for Copenhagen and Frederiksberg (KF 01 328 723) approved the study and all participants gave oral and written informed consent.

Magnetic resonance images (MRI) of the brain were acquired using a 3.0 Tesla Siemens Verio scanner with a 32-channel head coil (Siemens, Erlangen, Germany). Data from the same study cohort have been used in two related publications [17, 18] and will therefore be only shortly summarised. Two DWI data sets of the whole brain (61 axial slices with an isotropic voxel resolution of 2.3 mm) were consecutively acquired using a twice-refocused spin-echo sequence [19]. Sequence settings were as follows: TE = 89 ms, TR = 11440 ms, Grappa factor = 2, with 24 reference lines and 61 uniformly distributed directions with *b* = 1500 s/mm^2^, and 10 non-diffusion-weighted images. For postprocessing purposes, a set of non-diffusion-weighted reversed phase-encoding images was acquired with the same imaging parameters. High-resolution T1-weighted structural image volumes were acquired for anatomical information (TE = 2.32 ms, TR = 1900 ms, and flip angle = 9°) with an isotropic voxel resolution of 0.9 mm.

### 2.2. Data Preprocessing

We used MATLAB R2012a (MathWorks, Inc., Natick, Massachusetts, USA) and FSL [20–22] for image processing. Susceptibility artefacts in the raw DWIs were minimised with the application of a voxel displacement map (VDM). The two reversed phase-encoding *b* = 0 s/mm^2^ images were used for estimating the voxel shifts for the VDM, as implemented in FSL's topup tool [20, 23]. We applied the VDM and a full affine transformation to correct for movement and eddy currents in the DWIs using FSL's eddy tool [24], by reslicing to original image resolution using spline interpolation. Finally, the 61 directions were reoriented similarly to the orientation introduced by the applied transformations [25]. The T1-weighted images were corrected for gradient nonlinearities [26].

### 2.3. Defining Seed and Target Masks of Thalamus and Cortex

To examine the thalamocortical connectivity pattern, seed and target masks for tractography were defined. The target masks were obtained through a subject-based cortical segmentation with FreeSurfer software v. 5.3.0 [27, 28] based on the averaged T1-weighted volumes [29]. The cortical segmentation from FreeSurfer was used to define anatomical regions of interest using the Desikan-Killiany atlas [30]. We created five cortical target masks, which were used for tractography, including the occipital, temporal, somatosensory/parietal-postcentral, motor/precentral, and frontal cortical areas.  Table 1 shows the expected relation in terms of brain connectivity in the normal healthy human brain between the five cortical target masks and thalamic regions. The seed mask for tractography included all voxels within the thalamus region. This region also included the LGN and MGN as defined in the AAL atlas [31]. The seed mask was then transformed from MNI space into each individual subject's diffusion space for tractography. This was done using nonlinear registration from the MNI template to the structural T1-weighted image space and then using linear registration from the T1-weighted to the non-diffusion-weighted image. For each subject, the thalamic seed mask was manually edited to ensure proper coverage of the anatomical area of the thalamus. The cortical target masks were transformed from each individual subject in FreeSurfer space through the structural T1-weighted images to each individual subject's diffusion space through the FA image. The transformations were done using FreeSurfer and FSL v. 5.0 registration tools [20–22, 32, 33].

### 2.4. Tractography-Based Thalamocortical Segmentation

Based on the preprocessed DWIs, we used FSL's bedpostx tool to estimate the multifibre directions within each voxel of the brain using the ball-and-two-sticks model [34, 35] as the fibre orientation distribution function for subsequent tractography. For tractography-based thalamic segmentation, we used the tractography and clustering procedure available in FSL and standard settings [16]. First and in native space, probabilistic tractography was performed for each voxel in the thalamic seed mask (5000 streamlines per voxel) and to each of the five cortical target masks. Tractography was run separately in each hemisphere, and a midsagittal exclusion mask ensured exclusion of transhemispheric connections. Then, FSL “find-the-biggest” hard clustering approach was applied to the tractography results to segment the thalamus into five clusters, corresponding to the tractography connectivity pattern to each of the five cortical target masks. For each individual, seeds-to-target information on the connectivity from the seed to each individual target mask was obtained from the tractography. Based on this information, the find-the-biggest clustering assigns each voxel within the thalamus to the class with the highest connectivity count. We additionally used the seeds-to-target information as a marker of connection strength between the thalamus and each cortical target mask represented by the connectivity count within each voxel of the thalamus normalised with total number of streamlines. Lastly, to extract a region of interest within the individual thalamocortical white matter projections between the thalamus and each cortical target mask, tractography was run from the thalamus to each individual target mask, using the remaining cortical masks as exclusion masks.

### 2.5. Volume and DTI-Derived Indices of Whole Thalamus and Thalamic Clusters

The overall thalamic volume was assessed in native space based on the T1-weighted images. The volume of each cluster was evaluated in native diffusion image space based on the five cluster segmentations. The volume of each cluster was normalised with the total thalamic volume. Applying a diffusion tensor model to the preprocessed DWIs, the DTI-derived indices fractional anisotropy (FA) and mean diffusivity (MD) were extracted for each voxel and the mean value was calculated for the whole thalamus and each individual cluster.

### 2.6. Microstructural Features of Thalamocortical White Matter Projections

Regions of interest within the white matter projections from the thalamus to each cortical target mask were extracted from the tractography by thresholding. A threshold of 40% of the highest number of streamlines connecting the thalamus and the target was set to focus on the core tract area, that is, typically situated in the midline of the tract, removing spurious streamlines. Furthermore, we ensured that there was no overlap between white matter projections by keeping only the streamlines within the tract that had the highest connection strength. MD and FA were extracted to calculate the mean of each voxel within this core tract region of interest of each white matter projection.

### 2.7. Statistical Analyses

Based on our previous report on thalamic volume reductions in congenitally blind subjects [8], we applied a one-tailed *t*-test under the assumption of unequal variance between groups to test for between-group differences in overall thalamic volume at a significance level of *p* < 0.05. A one-way ANOVA was applied to test for differences in individual cluster volume, correcting for multiple comparisons using the Bonferroni method.

We used a similar one-way ANOVA for testing the overall group differences of the DTI-derived microstructural indices within the whole thalamus and thalamic clusters at a significance level of *p* < 0.05. Group and cluster were main effects, whereas “group × cluster” was the interaction of interest. Post hoc two-sample *t*-tests were used to assess differences between each group and cluster, adjusting for multiple testing. The same procedure was applied to test for statistical differences in MD and FA in the five white matter projections from each thalamic cluster. For all variables, Levene's test was used to check for homogeneity of the variance prior to application of the ANOVA.

## 3. Results

### 3.1. Connectivity-Based Segmentation of the Thalamus

**Figure 1:**
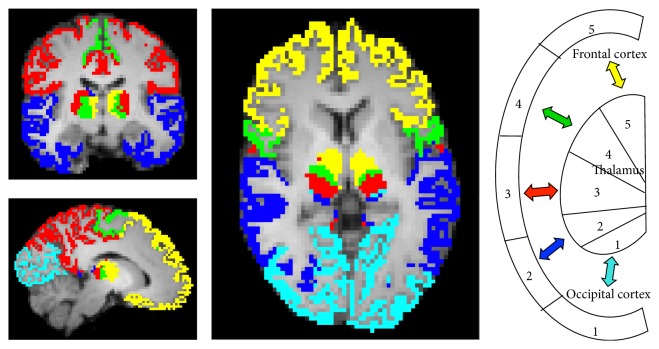
Find-the-biggest segmentation of the thalamus into five clusters and their corresponding cortical target masks in a NS subject (*x* = 52, *y* = 48, *z* = 28). Occipital cluster 1 (light blue), temporal cluster 2 (dark blue), somatosensory postcentral cluster 3 (red), motor precentral cluster 4 (green), and frontal cluster 5 (yellow). The right part of the figure shows a color-coded schematic illustration of the thalamocortical connectivity.

Connectivity-based segmentation of the thalamus resulted in five clusters.  Figure 1 shows the thalamic segmentation pattern and the corresponding cortical target masks in a normal sighted control. The occipital cluster (light blue) comprised mainly the LGN that projects to the primary visual cortex. The temporal thalamic cluster (dark blue) included mainly the MGN but also the pulvinar, which projects to occipital, parietal, and temporal areas. We wish to point out that because of the close anatomical proximity of the LGN and MGN in the metathalamus and overlapping thalamic projections, the clustering method is not likely to separate these small structures completely. The sensory thalamic cluster (red) included the lateral nucleus and the ventral posterior and the ventral lateral thalamic nuclei projecting to posterior parietal and postcentral cortical areas. The motor cluster (green) covered the ventral anterior nucleus projecting to precentral but also superior frontal cortices. The frontal cluster (yellow) included the anterior nucleus and the mediodorsal nucleus, which project to superior frontal and prefrontal areas.

**Figure 2:**
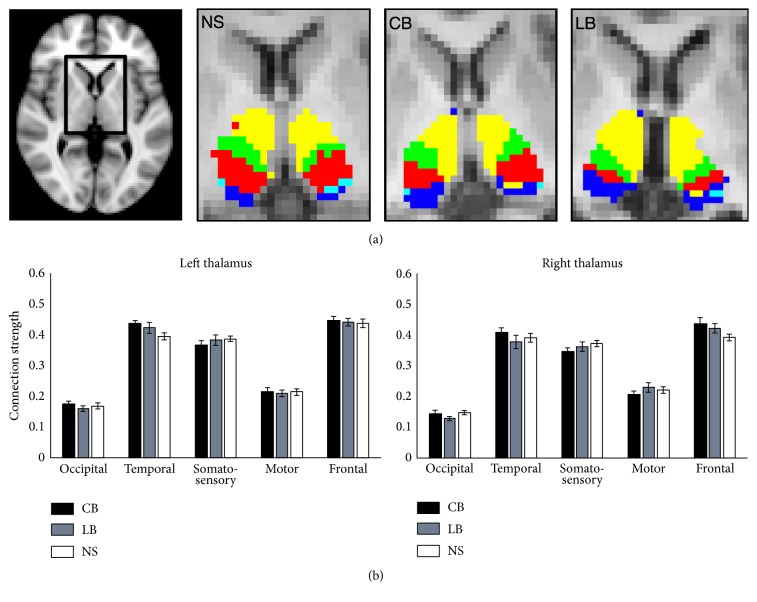
(a) Find-the-biggest segmentation within thalamus in a CB, LB, and NS participant. (b) Bar plot of the thalamocortical connectivity strength for the left and right thalamus for all CB, LB, and NS subjects. The *y*-axis represents the marker of connection strength between the thalamus and each cortical target mask, as measured by the seed to target connectivity count normalised with the total number of streamlines. Error bars indicate the standard error of the mean.

Segmentation of the thalamus based on structural thalamocortical connectivity resulted in similar clusters in all groups (Figure 2).  Figure 2(a) shows an example of the thalamic segmentation pattern for a CB, LB, and NS subject, whereas Figure 2(b) shows a bar plot of the marker of connection strength, represented as the normalised seed to target, for each of the thalamocortical projections. There was no significant group difference in the connection strength. Hence, we could not confirm our hypothesis that blindness would be associated with differences in thalamocortical connectivity relative to normally sighted controls. Specifically, we found no increase in connectivity strength between occipital cortex and somatosensory and auditory projection nuclei. For all groups, the occipital cluster was very small, and the connection strength was much lower compared to that of the other clusters (Figure 2(b)). In several cases, the occipital cluster was overruled by the larger projections from nearby regions, which lead to no occipital cluster using the find-the-biggest segmentation (number of instances of presence of an occipital cluster for each group: CB: left 5/12, right 3/12 subjects; LB: left 8/15, right 2/15 subjects; NS: left 9/12, right 9/15 subjects).

### 3.2. Thalamic Volume and Microstructure

Overall thalamic volumes, corrected for intracranial brain volume, were significantly lower in congenitally blind (left/right: 6206 ± 702 mm^3^/6460 ± 763 mm^3^, *p* = 0.033 and 0.027, resp.) and late blind (left/right: 6059 ± 578 mm^3^/6419 ± 659 mm^3^, *p* = 0.002 and 0.008, resp.) individuals compared to sighted controls (left/right: NS: 6661 ± 439 mm^3^/7014 ± 603 mm^3^). Analysis of the MD and FA within the total thalamus mask (including all five clustered regions) showed a significant group difference in FA for the left (*F* = 10.69, *p* < 0.0005) and right (*F* = 12.17, *p* < 0.0005) thalamus. Post hoc tests revealed that reduced FA in CB (left and right: *p*_corr._ < 0.0005) and LB (left: *p*_corr._ < 0.02; right: *p*_corr._ < 0.03) caused the difference. Analysis of each thalamic cluster showed that the reduced FA was located in the temporal, sensory, and frontal cluster (Table 2). No differences were found in MD within the total thalamus mask or in any of the clusters.

### 3.3. Microstructural Properties within Thalamocortical Projections

**Figure 3:**
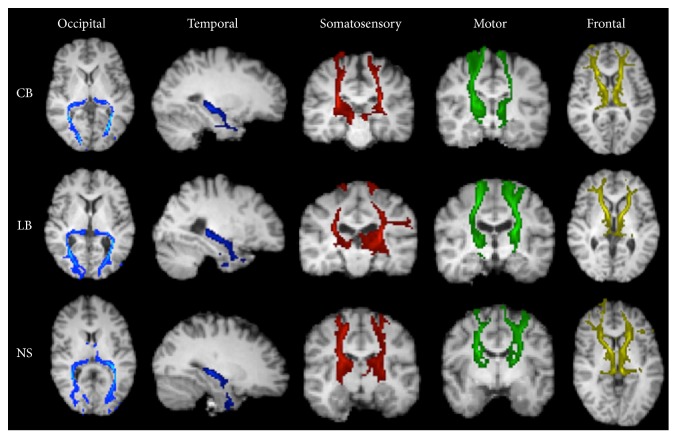
Examples of cortical white matter projections from each of the five thalamic clusters to the cortical target masks in a CB, LB, and NS participant. The cortical target masks are defined from the Desikan-Killiany atlas provided with FreeSurfer, as described in Table 1. The tracts are overlaid on each subject's T1-weighted image and presented in a slice of the most representative view, axial or coronal.

Figure 3 shows that the five thalamocortical white matter projections appeared visually similar across all three groups and also that the optic radiations remained present in the CB and LB groups, despite the absence of visual input (Figure 3, first column). MD and FA indices extracted within each of the five white matter projection regions of interest showed a significant group difference in FA for the occipital (left: *F* = 14.47, *p* < 0.0001; right: *F* = 25.19, *p* < 0.0001) and temporal (left: *F* = 7.58, *p* < 0.05; right: *F* = 7.19, *p* < 0.05) thalamocortical projections. Post hoc tests revealed reduced FA in CB and LB relative to the NS group (occipital left/right: *p*_corr._ < 0.0001; temporal left/right: *p*_corr._ < 0.05). A between-group difference was also found in MD for the occipital (left: *F* = 3.88, *p* < 0.05; right: *F* = 4.54, *p* < 0.05) and temporal (left: *F* = 3.65, *p* < 0.05; right: *F* = 3.66, *p* < 0.05) thalamic white matter projections. Post hoc tests revealed that this between-group difference was driven by an increased MD, but only in the LB group relative to the NS group (visual left/right: *p*_corr._ < 0.05; temporal left/right: *p*_corr._ < 0.05). No difference in MD was found in the CB group as compared to the NS group. FA and MD values within each white matter projection are shown in Table 3.

## 4. Discussion

We here present a connectivity-based clustering of the thalamus based on a hard segmentation between predefined cortical areas and the thalamus in blind and sighted individuals. We did not find evidence for changes in thalamocortical connectivity, as was shown with histological tracing techniques in animal studies (see [14]). Since DWI-based in vivo tractography maps larger fibre bundles and lacks specificity and sensitivity towards subtle axon bundles, these results do not rule out the possibility that there are subtle thalamocortical rearrangements related to blindness. However, analysis of DTI-derived microstructural indices revealed regional alterations in thalamic microstructure. Both blind groups showed reduced regional FA values relative to normal sighted controls in thalamic clusters that were connected to temporal, somatosensory, and frontal cortex. This indicates that blindness is associated with structural alterations within the thalamus and suggests a possible reorganisation of a thalamocortical connectivity pattern that was not captured with tractography. We also found a decrease in FA in the thalamooccipital and thalamotemporal projections. Furthermore, late blind, but not congenitally blind, participants additionally showed increased MD in these tracts compared to normal controls. However, these microstructural changes were not sufficiently large to be reflected in the tractography-based thalamocortical connectivity results. We suggest that changes in the microstructural environment are not a direct indicator of thalamic reorganisation but rather reflect a neuroplastic effect of the thalamic projections responding to a functional change. However, specific functional studies are needed to support this structural-functional relation in the blind.

### 4.1. How Does Nonvisual Information Reach the Visual Cortex?

The functional reorganisation of the visually deprived human brain could be supported either by unmasking of already existing thalamic projections or by the development of new connections. Changes in thalamocortical connectivity might be related to connections from nonvisual thalamic nuclei that send out new axon collaterals conveying nonvisual information flow to the visual cortex. Despite a complete lack of afferent visual input, the geniculocalcarine pathway remains relatively spared [11, 12, 36, 37]. Data from the present study confirm that the connectivity between the thalamus and the occipital cortex in the human is preserved in congenitally blind individuals, as testified by the absence of group differences in posterior thalamic clusters and normal looking optic radiations on visual inspection. Since congenitally blind individuals have never had any visual experience, it seems plausible that the geniculocalcarine tract is kept alive by the relay of nonvisual sensory information to the occipital cortex, a contention that is now generally accepted (see [38] for review). For instance, Bridge et al. [12] reported a decrease in FA of the optic radiations but suggested normal connectivity, assessed through the number of tractography streamlines (i.e., strength of connectivity) from the thalamus to the primary visual cortex in six anophthalmic subjects. Using anatomical connectivity mapping (ACM) and DTI, we also previously reported decreased FA within the optic radiations in the same blind groups, whereas ACM was mainly decreased within the splenium and midbody of the corpus callosum [18]. A retrograde tracer injection study in the primary visual cortex of early bilaterally enucleated opossums reported preservation of normal connectivity, but also new corticocortical and thalamocortical connections [14]. The authors suggested that the visually deprived occipital cortex receives inputs from various nonvisual thalamic nuclei related to somatosensory, auditory (see also [39]), and motor functions. Hamsters and ferrets, in which the occipital cortex has been ablated at birth, leaving the eyes and the optic tract intact, show a rearrangement of the thalamic intrinsic connectivity patterns [40]. Anatomical tracer studies in these animals have shown that the MGN becomes invaded by retinal projections and that neurons within the auditory cortex now also respond to visual stimuli. Importantly, rewired animals can use their auditory cortex to perform visual tasks (for review, see [38, 41]). Such thalamic rearrangements might result in a subtle shift in the border between thalamic nuclei projecting to auditory and visual areas, whereby projections from the MGN invade the LGN or the pulvinar nucleus, thereby rerouting auditory information to the visual cortex. Other studies have also reported a similar mechanism when the somatosensory thalamic nucleus (VB) receives projections from the retina [41]. VB neurons in rewired animals project to the somatosensory cortex and become responsive to visual stimulation [41].

Using tractography-based thalamic network analysis, we did not find evidence for a shift in the borders of the thalamic clusters, nor for a change in thalamocortical connectivity per se. However, our findings of decreased FA within several clusters of the thalamus suggest a change in the microstructural distribution of fibre connections within the thalamus, possibly related to a change in the internal connectivity between the clusters. We previously reported an overall volumetric reduction of the thalamus as well as several of its atlas-based subdivisions in congenitally blind subjects [8, 11, 41]. The present data confirm and extend these findings by showing that similar volumetric reductions take place in late blind individuals.

The reduction in the size of the thalamus, and particularly that of the LGN, parallels animal models of early visual deprivation, enucleation, or cortical lesions. For example, the early cortical ablation of all visual cortical areas in the monkey leads to a largely reduced dLGN that is still layered and metabolically active [42, 43].

### 4.2. The Link between Functional and Structural Reorganisation: Methodological Limitations

The animal and human literature on visual deprivation provides key elements that subcortical mechanisms play an important role in the reorganisation of the thalamofugal projections [38, 41]. In animal models, subcortical rearrangements lead to rerouting of the auditory and somatosensory inputs to the dLGN [13, 14, 41]. Using dynamic causal modelling analysis of human fMRI data, Klinge et al. [44] argue in favour of increased corticocortical connectivity, supporting the hypothesis of increased functional connectivity between the primary auditory and primary visual cortex, as opposed to increased thalamocortical connectivity. There is also evidence for increased corticocortical connectivity between primary somatosensory and occipital cortex in congenital blindness of humans, as demonstrated by transcranial magnetic stimulation (TMS) [15], combined TMS and positron emission tomography [45], and magnetoencephalography studies [4]. Behavioral and imaging studies using a sensory substitution device that translates visual information into electrotactile stimulation also showed increased corticocortical connectivity between the primary somatosensory and primary visual cortex [3, 15]. However, the strengthening of corticocortical connectivity does not preclude the possibility that changes also may take place in thalamocortical connectivity, and the two types of connectivity changes may exist together. Interestingly, we did observe microstructural changes along some thalamocortical connections, suggesting that connectivity-related changes have taken place, but not at a level reflected in the thalamocortical connectivity revealed by tractography.

We found a difference in mean FA within the occipital and temporal white matter projections in both groups of blind subjects. Even though we restricted the region of interest to the core part of the tracts for voxel-wise analysis, differences in mean FA can be caused by the modulation of macroscopic effects, such as bending, crossing, and fanning axons [46]. Macrostructural effects as tract shape and volume may blur true differences in microscopic anisotropy [47] in blind individuals.

We selected a constrained thalamocortical segmentation method, based on five predefined cortical areas that are connected with different thalamic nuclei. Although the five-cluster segmentation gave robust thalamic clusters, it grouped several thalamic nuclei into one common cluster, making it difficult to distinguish visual, auditory, and somatosensory connectivity. Furthermore, the find-the-biggest approach is a hard clustering approach not taking into account less probable connections in the final segmentation. However, the find-the-biggest approach is based on the “seeds-to-target” analysis, in which each voxel is given a number according to the probability of that voxel to be assigned to a certain cortical mask. This hard clustering approach does not allow an examination of potential reorganisations within the cortex. Modulation of the thalamocortical projections might also be reflected at the cortical level through internal corticocortical connectivity changes. Alternatively, an unconstrained clustering method, such as *k*-means clustering, could be used. Our exploratory results using this unconstrained method (data not shown) demonstrate that five clusters is a good choice, supporting the constrained method used [16, 48, 49]. However, unconstrained clustering provides noisy results, challenging a robust definition of thalamic clusters. Different sources can contribute to the noisy clustering results, for instance, seeding streamlines within grey matter voxels. Since subcortical grey matter areas as the thalamus have low anisotropy, this introduces fluctuations in tractography streamlining until they reach the surface of thalamus and enter the white matter tracts. These fluctuations can introduce false positives as well as false negatives of especially the finer detailed tracts and therefore mainly the gross tract systems are reliably estimated with tractography [50, 51]. When using constrained clustering with fixed cortical regions, we reduce the noise but at the cost of losing finer connectivity details. However, to support the robustness of finer tracking details, that is, white matter tracts consisting of fewer numbers of streamlines, the data must be supplemented by results of tracer studies in animals and translated to human in combination with Klingler dissection [52, 53].

## 5. Conclusion

We here presented a connectivity-based segmentation of the thalamus into five clusters and showed differences in DTI indices within the thalamus in congenital and late-onset blindness. These results indicate overall preservation of the structural thalamocortical connectivity but suggest that a reorganisation has taken place at the level of the thalamic nuclei. However, changes in DTI indices did appear in some of the thalamocortical projections, but they did not impact the overall structural thalamocortical network as revealed by tractography. The absence of macrostructural changes might be explained by a combination of how the tractography works and image quality (i.e., signal-to-noise and image resolution). The use of high-field MRI at 7 teslas might furnish a more appropriate tool for the detailed investigation of the connectivity pattern within the thalamus and cortex in sensory deprivation.

## Figures and Tables

**Table 1:** Overview of the cortical target masks and the corresponding thalamic nuclei.

Target mask	Cortical labels	Corresponding thalamic nuclei
Occipital	Pericalcarine Cuneus Lateral occipital Lingual	Pulvinar LGN MGN Lateral nucleus

Temporal	Middle temporal Inferior temporal Superior temporal Transverse temporal Fusiform Temporal pole Banks of superior temporal sulcus Entorhinal Parahippocampal	Pulvinar MGN

Somatosensory/parietal-postcentral	Postcentral Supramarginal Superior parietal Inferior parietal Precuneus Isthmus cingulate Posterior cingulate	Lateral nucleus Ventrolateral nucleus Ventroposterior nucleus

Motor/precentral	Precentral Caudal middle frontal Paracentral	Ventrolateral nucleus Ventroanterior nucleus

Frontal	Superior frontal Frontal pole Medial orbitofrontal Lateral orbitofrontal Rostral middle frontal Rostral anterior cingulate Pars opercularis Pars orbitalis Pars triangularis	Anterior nucleus Mediodorsal nucleus

Cortical target masks defined by combining labels given by the Desikan-Killiany atlas. The last column shows the thalamic nuclei, corresponding to the respective cortical projection sites as defined from an anatomical atlas based on the normal healthy human brain [5]. LGN: lateral geniculate nuclei; MGN: medial geniculate nuclei.

**Table 2:** Mean and standard deviations of FA within the whole thalamus mask and the five segmented clusters.

		Thalamus			Occipital			Temporal			Sensory			Motor			Frontal		
		CB	LB	NS	CB	LB	NS	CB	LB	NS	CB	LB	NS	CB	LB	NS	CB	LB	NS
FA Mean (SD)	L	0.28^*∗∗*^	0.29^*∗*^	0.31	0.31	0.29	0.30	0.26^*∗∗*^	0.27	0.29	0.30^*∗*^	0.32	0.33	0.34	0.35	0.38	0.26^*∗∗*^	0.27^*∗*^	0.29
		(0.02)	(0.02)	(0.02)	(0.07)	(0.05)	(0.03)	(0.03)	(0.02)	(0.02)	(0.03)	(0.03)	(0.02)	(0.05)	(0.06)	(0.01)	(0.02)	(0.02)	(0.02)
	R	0.28^*∗∗*^	0.30^*∗*^	0.32	0.28	0.28	0.31	0.24^*∗∗*^	0.26^*∗*^	0.29	0.31^*∗*^	0.33	0.35	0.36	0.36	0.38	0.26^*∗∗*^	0.28	0.29
		(0.03)	(0.02)	(0.02)	(0.07)	(0.10)	(0.06)	(0.03)	(0.03)	(0.02)	(0.04)	(0.03)	(0.02)	(0.05)	(0.04)	(0.04)	(0.03)	(0.02)	(0.02)

Significantly reduced FA in CB and LB compared to NS at ^*∗∗*^*p* < 0.001 or ^*∗*^*p* < 0.05, Bonferroni corrected. CB: congenitally blind; LB: late blind; NS: normal sighted controls; L: left; R: right; FA: fractional anisotropy; SD: standard deviation.

**Table 3:** Mean and standard deviations of FA and MD within the five thalamic white matter projections.

		Occipital			Temporal			Sensory			Motor			Frontal		
		CB	LB	NS	CB	LB	NS	CB	LB	NS	CB	LB	NS	CB	LB	NS
FA Mean (SD)	L	0.46^*∗∗*^	0.45^*∗∗*^	0.53	0.36^*∗*^	0.35^*∗*^	0.40	0.45	0.44	0.45	0.47	0.43	0.44	0.41	0.41	0.41
		(0.05)	(0.05)	(0.03)	(0.05)	(0.04)	(0.02)	(0.05)	(0.03)	(0.04)	(0.04)	(0.05)	(0.05)	(0.05)	(0.03)	(0.04)
	R	0.43^*∗∗*^	0.42^*∗∗*^	0.51	0.35^*∗*^	0.34^*∗*^	0.39	0.44	0.44	0.44	0.46	0.44	0.46	0.40	0.38	0.40
		(0.05)	(0.04)	(0.03)	(0.04)	(0.04)	(0.03)	(0.04)	(0.03)	(0.04)	(0.03)	(0.03)	(0.04)	(0.04)	(0.03)	(0.03)

MD Mean (SD)	L	0.67	0.72^*∗*^	0.65	0.71	0.72^*∗*^	0.67	0.64	0.64	0.64	0.63	0.65	0.65	0.62	0.62	0.62
		(0.04)	(0.09)	(0.04)	(0.04)	(0.07)	(0.04)	(0.04)	(0.05)	(0.06)	(0.06)	(0.08)	(0.09)	(0.04)	(0.05)	(0.03)
	R	0.70	0.71^*∗*^	0.64	0.71	0.72^*∗*^	0.67	0.62	0.62	0.62	0.61	0.62	0.61	0.64	0.65	0.64
		(0.05)	(0.09)	(0.03)	(0.04)	(0.06)	(0.04)	(0.03)	(0.04)	(0.04)	(0.04)	(0.05)	(0.04)	(0.04)	(0.05)	(0.02)

Significantly reduced FA in CB and LB and increased MD in LB compared to NS at ^*∗∗*^*p* < 0.001 or ^*∗*^*p* < 0.05, Bonferroni corrected. CB: congenitally blind; LB: late blind; NS: normal sighted controls; L: left; R: right; FA: fractional anisotropy; MD: mean diffusivity, values × 10^−3^; SD: standard deviation.

## References

[1] Kupers R., Ptito M. (2011). Insights from darkness: what the study of blindness has taught us about brain structure and function. *Progress in Brain Research*.

[2] Bavelier D., Neville H. J. (2002). Cross-modal plasticity: where and how?. *Nature Reviews Neuroscience*.

[3] Ptito M., Moesgaard S. M., Gjedde A., Kupers R. (2005). Cross-modal plasticity revealed by electrotactile stimulation of the tongue in the congenitally blind. *Brain*.

[4] Ioannides A. A., Liu L., Poghosyan V. (2013). MEG reveals a fast pathway from somatosensory cortex to occipital areas via posterior parietal cortex in a blind subject. *Frontiers in Human Neuroscience*.

[5] Nieuwenhuys R., Voogd J., van Huijzen C. (1988). *The Human Central Nervous System*.

[6] Amaral D. G., Kandel E. R., Schwartz J. H., Jessel T. M., Siegelbaum S. A., Hudspeth A. J. (2013). The functional organisation of perception and movement. *Principles of Neural Science*.

[7] Metzger C. D., Van der Werf Y. D., Walter M. (2013). Functional mapping of thalamic nuclei and their integration into cortico-striatal-thalamo-cortical loops via ultra-high resolution imaging—*from animal anatomy to in vivo imaging in humans*. *Frontiers in Neuroscience*.

[8] Cecchetti L., Ricciardi E., Handjaras G., Kupers R., Ptito M., Pietrini P. (2016). Congenital blindness affects diencephalic but not mesencephalic structures in the human brain. *Brain Structure and Function*.

[9] Noppeney U., Friston K. J., Ashburner J., Frackowiak R., Price C. J. (2005). Early visual deprivation induces structural plasticity in gray and white matter. *Current Biology*.

[10] Pan W.-J., Wu G., Li C.-X., Lin F., Sun J., Lei H. (2007). Progressive atrophy in the optic pathway and visual cortex of early blind Chinese adults: a voxel-based morphometry magnetic resonance imaging study. *NeuroImage*.

[11] Ptito M., Schneider F. C. G., Paulson O. B., Kupers R. (2008). Alterations of the visual pathways in congenital blindness. *Experimental Brain Research*.

[12] Bridge H., Cowey A., Ragge N., Watkins K. (2009). Imaging studies in congenital anophthalmia reveal preservation of brain architecture in 'visual' cortex. *Brain*.

[13] Hyvärinen J., Hyvärinen L., Linnankoski I. (1981). Modification of parietal association cortex and functional blindness after binocular deprivation in young monkeys. *Experimental Brain Research*.

[14] Karlen S. J., Kahn D. M., Krubitzer L. (2006). Early blindness results in abnormal corticocortical and thalamocortical connections. *Neuroscience*.

[15] Kupers R., Fumal A., De Noordhout A. M., Gjedde A., Schoenen J., Ptito M. (2006). Transcranial magnetic stimulation of the visual cortex induces somatotopically organized qualia in blind subjects. *Proceedings of the National Academy of Sciences of the United States of America*.

[16] Behrens T. E. J., Johansen-Berg H., Woolrich M. W. (2003). Non-invasive mapping of connections between human thalamus and cortex using diffusion imaging. *Nature Neuroscience*.

[17] Reislev N. L., Kupers R., Siebner H. R., Ptito M., Dyrby T. B. (2016). Blindness alters the microstructure of the ventral but not the dorsal visual stream. *Brain Structure and Function*.

[18] Reislev N. L., Dyrby T. B., Siebner H. R., Kupers R., Ptito M. (2016). Simultaneous assessment of white matter changes in microstructure and connectedness in the blind brain. *Neural Plasticity*.

[19] Reese T. G., Heid O., Weisskoff R. M., Wedeen V. J. (2003). Reduction of eddy-current-induced distortion in diffusion MRI using a twice-refocused spin echo. *Magnetic Resonance in Medicine*.

[20] Smith S. M., Jenkinson M., Woolrich M. W. (2004). Advances in functional and structural MR image analysis and implementation as FSL. *NeuroImage*.

[21] Woolrich M. W., Jbabdi S., Patenaude B. (2009). Bayesian analysis of neuroimaging data in FSL. *NeuroImage*.

[22] Jenkinson M., Beckmann C. F., Behrens T. E. J., Woolrich M. W., Smith S. M. (2012). FSL. *NeuroImage*.

[23] Andersson J. L. R., Skare S., Ashburner J. (2003). How to correct susceptibility distortions in spin-echo echo-planar images: application to diffusion tensor imaging. *NeuroImage*.

[24] Andersson J. L. R., Sotiropoulos S. N. (2015). An integrated approach to correction for off-resonance effects and subject movement in diffusion MR imaging. *NeuroImage*.

[25] Alexander D. C., Pierpaoli C., Basser P. J., Gee J. C. (2001). Spatial transformations of diffusion tensor magnetic resonance images. *IEEE Transactions on Medical Imaging*.

[26] Jovicich J., Czanner S., Greve D. (2006). Reliability in multi-site structural MRI studies: effects of gradient non-linearity correction on phantom and human data. *NeuroImage*.

[27] Dale A. M., Fischl B., Sereno M. I. (1999). Cortical surface-based analysis: I. Segmentation and surface reconstruction. *NeuroImage*.

[28] Fischl B., Sereno M. I., Dale A. M. (1999). Cortical surface-based analysis: II. Inflation, flattening, and a surface-based coordinate system. *NeuroImage*.

[29] Reuter M., Rosas H. D., Fischl B. (2010). Highly accurate inverse consistent registration: a robust approach. *NeuroImage*.

[30] Desikan R. S., Ségonne F., Fischl B. (2006). An automated labeling system for subdividing the human cerebral cortex on MRI scans into gyral based regions of interest. *NeuroImage*.

[31] Tzourio-Mazoyer N., Landeau B., Papathanassiou D. (2002). Automated anatomical labeling of activations in SPM using a macroscopic anatomical parcellation of the MNI MRI single-subject brain. *NeuroImage*.

[32] Jenkinson M., Smith S. (2001). A global optimisation method for robust affine registration of brain images. *Medical Image Analysis*.

[33] Jenkinson M., Bannister P., Brady M., Smith S. (2002). Improved optimization for the robust and accurate linear registration and motion correction of brain images. *NeuroImage*.

[34] Behrens T. E. J., Woolrich M. W., Jenkinson M. (2003). Characterization and propagation of uncertainty in diffusion-weighted MR imaging. *Magnetic Resonance in Medicine*.

[35] Behrens T. E. J., Berg H. J., Jbabdi S., Rushworth M. F. S., Woolrich M. W. (2007). Probabilistic diffusion tractography with multiple fibre orientations: what can we gain?. *NeuroImage*.

[36] Shu N., Liu Y., Li J., Li Y., Yu C., Jiang T. (2009). Altered anatomical network in early blindness revealed by diffusion tensor tractography. *PLoS ONE*.

[37] Wang D., Qin W., Liu Y., Zhang Y., Jiang T., Yu C. (2013). Altered white matter integrity in the congenital and late blind people. *Neural Plasticity*.

[38] Kupers R., Ptito M. (2014). Compensatory plasticity and cross-modal reorganization following early visual deprivation. *Neuroscience and Biobehavioral Reviews*.

[39] Laemle L. K., Strominger N. L., Carpenter D. O. (2006). Cross-modal innervation of primary visual cortex by auditory fibers in congenitally anophthalmic mice. *Neuroscience Letters*.

[40] Ptito M., Giguère J.-F., Boire D., Frost D. O., Casanova C. (2001). When the auditory cortex turns visual. *Progress in Brain Research*.

[41] Desgent S., Ptito M. (2012). Cortical GABAergic interneurons in cross-modal plasticity following early blindness. *Neural Plasticity*.

[42] Boire D., Théoret H., Ptito M. (2001). Visual pathways following cerebral hemispherectomy. *Progress in Brain Research*.

[43] Ptito M., Herbin M., Boire D., Ptito A. (1996). Neural bases of residual vision in hemicorticectomized monkeys. *Progress in Brain Research*.

[44] Klinge C., Eippert F., Röder B., Büchel C. (2010). Corticocortical connections mediate primary visual cortex responses to auditory stimulation in the blind. *Journal of Neuroscience*.

[45] Wittenberg G. F., Werhahn K. J., Wassermann E. M., Herscovitch P., Cohen L. G. (2004). Functional connectivity between somatosensory and visual cortex in early blind humans. *European Journal of Neuroscience*.

[46] Jespersen S. N., Lundell H., Sønderby C. K., Dyrby T. B. (2013). Orientationally invariant metrics of apparent compartment eccentricity from double pulsed field gradient diffusion experiments. *NMR in Biomedicine*.

[47] Vos S. B., Jones D. K., Jeurissen B., Viergever M. A., Leemans A. (2012). The influence of complex white matter architecture on the mean diffusivity in diffusion tensor MRI of the human brain. *NeuroImage*.

[48] Johansen-Berg H., Behrens T. E. J., Sillery E. (2005). Functional-anatomical validation and individual variation of diffusion tractography-based segmentation of the human thalamus. *Cerebral Cortex*.

[49] Zhang D., Snyder A. Z., Shimony J. S., Fox M. D., Raichle M. E. (2010). Noninvasive functional and structural connectivity mapping of the human thalamocortical system. *Cerebral Cortex*.

[50] Dyrby T. B., Søgaard L. V., Parker G. J. (2007). Validation of in vitro probabilistic tractography. *NeuroImage*.

[51] Thomas C., Ye F. Q., Irfanoglu M. O. (2014). Anatomical accuracy of brain connections derived from diffusion MRI tractography is inherently limited. *Proceedings of the National Academy of Sciences of the United States of America*.

[52] Innocenti G. M., Dyrby T. B., Andersen K. W., Rouiller E. M., Caminiti R. (2016). The crossed projection to the striatum in two species of monkey and in humans: behavioral and evolutionary significance. *Cerebral Cortex*.

[53] Hau J., Sarubbo S., Houde J. C. (2016). Revisiting the human uncinate fasciculus, its subcomponents and asymmetries with stem-based tractography and microdissection validation. *Brain Structure and Function*.

